# Supplementary material for: The non-avian theropod quadrate I: standardized terminology with an overview of the anatomy and function
Source: PeerJ. 2015 Sep 17;3:e1245. doi: 10.7717/peerj.1245 (PMC4579021; doi:10.7717/peerj.1245)
Supplement: Supplemental Information 1 — Function of quadrate sub-entities, quadrate sub-units terminology, and quadrate ontogeny in Lourinhanosaurus autunesi and Shuvuuia deserti. [file peerj-03-1245-s001.docx]

# Supplemental information

Appendix 1: Function of quadrate sub-entities.

Appendix 2: Quadrate sub-units terminology.

References

## Appendix 1. Function of quadrate sub-entities.

| **Anatomical component** | **Nature** | **Anatomical sub-entity** | **Function** | **Theropod clades** | **Publication** |
| --- | --- | --- | --- | --- | --- |
| Quadrate diverticulum | Tympanic sinus, mandibular arch pneumatic system | Anterior, posterior, medial, ventral and dorsal pneumatic foramina | Auditory function? | All Theropoda | (Witmer 1990; Kundrát and Janáček 2007; Tahara and Larsson 2011) |
| Siphoneal diverticulum | Tympanic sinus, mandibular arch pneumatic system | Ventral pneumatic foramina? | Auditory function? | Allosauroidea?  Tyrannosauroidea? | (Tahara and Larsson 2011) |
| Dorsal tympanic diverticulum | Tympanic sinus | Quadrate head, separated into otic and squamosal capitula | Auditory function | Neognathae | (Witmer 1990) |
| Musculus Protractor Pterygoideus (MPPt) | Orbitotemporal muscle | Medial surface of the pterygoid flange | Insertion for the MPPt | Avian and non-avian? Theropoda | (Holliday 2009) |
| Musculus Adductor Mandibulae Externus Medialis (MAMEM) + Musculus Adductor Mandibulae Externus Superficialis (MAMES) | Temporal  muscle | Posteromedial portion of the quadrate head | Origin for the MAMEM + MAMES | Avian and non-avian? Theropoda | (Sakamoto 2008) |
| Musculus Adductor Mandibulae  Posterior (MAMP) | Palatal  muscle | Lateral surface of the pterygoid flange | Origin for the MAMP | All Theropoda | (Molnar 1998; Sakamoto 2008; Holliday 2009) |
| Musculus Pseudotemporalis Profundus (MPsTP) | Palatal  muscle | Anterior surface of the quadrate head | Origin for the MPsTP | Neornithes | (Holliday and Witmer 2008; Sakamoto 2008; Holliday 2009) |
| Otic joint | Synovial joint | Quadrate head | Articulation with squamosal | All Theropoda | (Holliday and Witmer 2008) |
| Articular process of quadrate | Synovial joint | Mandibular condyles | Articulation with mandible | All Theropoda | (Holliday and Witmer 2008) |
| Intercondylar sulcus | Synovial joint | Groove between ectocondyle and entocondyle | Ventral or ventrolateral displacement of mandible rami | All Theropoda | (Molnar 1991; Bakker 1998; Hendrickx and Buffetaut 2008) |
| Branches of maxillomandibular vessels | Neurovascular bundle | Quadrate foramen | Neurovasculature transmission between the occiput and adductor chamber | Some Theropoda | (Sampson and Witmer 2007) |

## Appendix 2. Terminology of nonavian theropod quadrate sub-unit by authors.

| **Source** | **Referred taxa** | **Quadrate shaft** | **Quadrate ridge** | **Pterygoid flange** | **Lateral process** | **Quadrate foramen** | **Quadrate head** | **Ento-/Ectocondyles** |
| --- | --- | --- | --- | --- | --- | --- | --- | --- |
| Sereno and Novas 1994 | *Herrerasaurus* | Shaft | / | Pterygoid ramus | / | Quadrate foramen | Head | Distal condyles |
| Colbert 1989 | *Coelophysis* | Ascending process | / | Quadrate flange | Smaller surface/wing | / | Upper extremity | Quadrate condyle |
| Welles 1984 | *Dilophosaurus* | Shaft | Column | Pterygoid wing | Dorsal wing | Quadrate foramen | Head | Ento/Ectocondyles |
| Smith et al. 2007 | *Cryolophosaurus* | Body | Rounded ridge | / | / | Paraquadrate fenestra | Dorsal head | Medial/Lateral condyles |
| Gilmore 1920 | *Ceratosaurus/*  *Allosaurus* | / | / | Sheet/projection | / | Quadrate foramen | / | articular surfaces |
| Madsen and Welles 2000 | *Ceratosaurus* | Pillar | / | Pterygoid wing | Anterolateral wing | Quadrate foramen | Head | Ento/Ectocondyles |
| Carrano et al. 2011 | *Masiakasaurus* | Shaft/body | / | Pterygoid articular flange | / | Foramen | / | Medial/Lateral condyles |
| Coria and Salgado 1998 | *Ilokelesia* | / | / | Medial process | Lateral lamina/process | / | Squamosal condyle | Medial/Lateral condyles |
| Bonaparte et al. 1990 | *Carnotaurus* | / | / | Anteromedial projection | / | / | / | Lower condyles |
| Sampson and Witmer 2007 | *Majungasaurus* | Shaft | / | Pterygoid ramus | Lateral/quadratojugal ramus | Paraquadrate foramen | Head | Medial/Lateral condyles |
| Sadleir et al. 2008 | *Eustreptospondylus* | Shaft | / | Pterygoid ala | / | / | Head | Medial/Lateral condyles |
| Britt 1991 | *Torvosaurus* | Shaft | / | Pterygoid blade | / | Quadrate foramen | Head | Medial/Lateral condyles |
| Charig and Milner 1997 | *Baryonyx* | Shaft | / | Pterygoid flange | / | Quadrate foramen | Head | Quadrate condyle |
| Madsen 1976 | *Allosaurus* | Shaft/body | / | Contact with the pterygoid | / | Quadrate foramen | Head | Condyles |
| Sereno et al. 2008 | *Aerosteon* | Shaft | / | Pterygoid process | / | Quadrate foramen | Head | Distal condyles |
| Currie 2006 | *Sinraptor* | Column | / | Pterygoid flange/ala | Wing-like process | Quadratic foramen | Quadrate cotyle | Medial/Lateral condyles |
| Currie and Carpenter 2000 | *Acrocanthosaurus* | / | / | / | / | Quadrate foramen | Head | Condyles |
| Eddy and Clarke 2011 | *Acrocanthosaurus* | / | / | Pterygoid wing/contact | / | Quadrate foramen/fenestra | Quadrate cotylus | Medial/Lateral condyles |
| Brusatte et al. 2010 | *Shaochilong* | Shaft | / | Quadrate flange | / | Quadrate foramen | Quadrate cotylus/head | Medial/Lateral condyles |
| Coria and Currie 2006 | *Mapusaurus* | / | / | Pterygoid flange | / | Quadratic foramen | Quadrate cotyle | Mandibular articulation |
| Choiniere et al. 2014*b* | *Aorun zhaoi* | Shaft, Quadrate shaft | / | Pterygoid ramus | / | Quadrate foramen | Quadrate head | Quadrate condyles |
| Rauhut et al. 2010 | *Proceratosaurus* | / | Columnar ridge | Pterygoid flange | / | Quadrate foramen | Head | Medial/Lateral condyles |
| Li et al. 2010 | *Xiongguanlong* | Shaft | / | Quadrate wing | / | Quadrate foramen | / | Medial/Lateral condyles |
| Brusatte et al. 2012 | *Alioramus* | Shaft | Robust ridge | Quadrate flange | / | Quadrate foramen | Head | Medial/Lateral condyles |
| Carr 1996 | *Albertosaurus* | Quadrate body | Ridge | Pterygoid flange/process | / | Quadrate fenestra | Quadrate cotyle | Medial/Lateral condyles |
| Currie 2003 | *Albertosaurus/*  *Daspletosaurus* | / | / | Pterygoid ala | / | Quadrate fenestra | Quadrate cotylus | Mandibular condyles |
| Molnar 1991 | *Tyrannosaurus* | Vertical bar | / | Pterygoid process | / | Quadrate foramen | Hemispherical surface | Quadrate condyle |
| Brochu 2003 | *Tyrannosaurus* | / | / | Pterygoidal flange | / | Quadrate foramen | Head | Medial/Lateral hemicondyles |
| Dal Sasso and Maganuco 2011 | *Scipionyx samniticus* | / | / | Pterygoid ala | / | Paraquadrate foramen | Quadrate head | Medial/Lateral condyles |
| Choiniere et al. 2014*a* | *Haplocheirus sollers* | Quadrate shaft | Ridge, pillar | Pterygoid ramus | / | Quadrate foramen | Quadrate head | Medial/Lateral condyles |
| Kobayashi and Lü 2003 | *Sinornithomimus* | / | / | Pterygoid wing | / | Paraquadratic foramen | / | Medial/Lateral condyles |
| Kobayashi and Barsbold 2005 | *Garudimimus* | / | / | Pterygoid wing | / | Paraquadratic foramen | / | Mandibular condyles |
| Zanno 2010 | *Falcarius* | / | / | Pterygoid process/flange/wing | / | Quadrate foramen | Head/Squamosal capitulum | Medial/Lateral condyles |
| Clark et al. 1994 | *Erlikosaurus* | Body | / | Orbital process | / | Quadrate foramen | Dorsal process | Mandibular process |
| Lautenschlager et al. 2014 | *Erlikosaurus* | Main body | Ridge | Pterygoid flange | Spike-like projection | Paraquadrate foramen | Quadrate head | Mandibular articulation (Medial/Lateral condyles) |
| Maryańska and Osmólska 1997 | *Oviraptoridae* | Shaft | / | Pterygoid ramus | / | Quadrate foramen | Otic process | Medial/Lateral condyles |
| Choiniere et al. 2010 | *Zuolong* | Shaft | Ridge | Pterygoid ramus | / | Quadrate foramen | Dorsal condyle | Medial/Lateral condyles |
| Burnham 2004 | *Bambiraptor* | / | / | / | / | / | Otic process | Medial/Lateral condyles |
| Norell et al. 2006 | *Tsaagan* | Shaft | / | Anterior/pterygoid flange | Squamosal ramus | Quadrate foramen | Dorsal articulation | Articular ramus |
| Colbert and Russell 1969 | *Dromaeosaurus* | Shaft |  | Pterygoid flange/wing | / | Quadrate foramen | Head | Medial/Lateral condyles |
| Currie 1995 | *Dromaeosaurus* | / |  | Anterodorsal lamina | / | Quadrate fenestra | Head | / |
| Norell and Hwang 2004 | *Saurornithoides* | Shaft |  | Pterygoid flange | / | / | / | Medial/Lateral condyles |
| Norell et al. 2009 | *Zanabazar* | Shaft |  | / | / | / | Head | / |

## References

Bakker, R. T. 1998. Brontosaur killers: late Jurassic allosaurids as sabre-tooth cat analogues. *Gaia* 15: 145–158.

Bonaparte, J. F., Novas, F. E. and Coria, R. A. 1990. *Carnotaurus sastrei* Bonaparte, the horned, lightly built carnosaur from the Middle Cretaceous of Patagonia. *Natural History Museum of Los Angeles County Contributions in Science* 416 (416): 1–42.

Britt, B. B. 1991. Theropods of Dry Mesa Quarry (Morrison Formation, Late Jurassic), Colorado, with emphasis on the osteology of *Torvosaurus tanneri*. *Brigham Young University Geology Studies* 37: 1–72.

Brochu, C. A. 2003. Osteology of *Tyrannosaurus rex*: Insights from a nearly complete skeleton and high-resolution computed tomographic analysis of the skull. *Journal of Vertebrate Paleontology* 22 (sup4): 1–138.

Brusatte, S. L., Carr, T. D. and Norell, M. A. 2012. The osteology of *Alioramus*, a gracile and long-snouted tyrannosaurid (Dinosauria: Theropoda) from the Late Cretaceous of Mongolia. *Bulletin of the American Museum of Natural History* 366: 1–197.

Brusatte, S. L., Chure, D. J., Benson, R. B. J. and Xu, X. 2010. The osteology of *Shaochilong maortuensis*, a carcharodontosaurid (Dinosauria: Theropoda) from the Late Cretaceous of Asia. *Zootaxa* 2334: 1–46.

Burnham, D. A. 2004. New Information on *Bambiraptor feinbergi* (Theropoda: Dromaeosauridae) from the Late Cretaceous of Montana. *In*: Currie, P. J., Koppelhus, E. B., Shugar, M. A. and Wright, J. L. (eds.), *Feathered Dragons: Studies on the Transition from Dinosaurs to Birds*, 67–111. Indiana University Press, Bloomington, Indiana.

Carrano, M. T., Loewen, M. A. and Sertich, J. J. W. 2011. New materials of *Masiakasaurus knopfleri* Sampson, Carrano, and Forster, 2001, and implications for the morphology of the Noasauridae (Theropoda: Ceratosauria). *Smithsonian Contributions to Paleobiology* 95: 1–53.

Carr, T. D. 1996. Cranial osteology and craniofacial ontogeny of Tyrannosauridae (Dinosauria: Theropoda) from the Dinosaur Park Formation (Judith River Group, Upper Cretaceous, Campanian) of Alberta. MSc. Dissertation, University of Toronto, Toronto, Ontario, Canada, 358pp.

Charig, A. J. and Milner, A. C. 1997. *Baryonyx walkeri*, a fish-eating dinosaur from the Wealden of Surrey. *Bulletin of the Natural History Museum* 53 (1): 11–70.

Choiniere, J. N., Clark, J. M., Forster, C. A. and Xu, X. 2010. A basal coelurosaur (Dinosauria: Theropoda) from the Late Jurassic (Oxfordian) of the Shishugou Formation in Wucaiwan, People’s Republic of China. *Journal of Vertebrate Paleontology* 30 (6): 1773–1796.

Choiniere, J. N., Clark, J. M., Norell, M. A. and Xu, X. 2014a. Cranial osteology of *Haplocheirus sollers* Choiniere et al., 2010 (Theropoda, Alvarezsauroidea). *American Museum Novitates* 3816.

Choiniere, J. N., Clark, J. M., Forster, C. A., Norell, M. A., Eberth, D. A., Erickson, G. M., Chu, H. and Xu, X. 2014b. A juvenile specimen of a new coelurosaur (Dinosauria: Theropoda) from the Middle–Late Jurassic Shishugou Formation of Xinjiang, People’s Republic of China. *Journal of Systematic Palaeontology* 12 (2): 177–215.

Clark, J. M., Perle, A. and Norell, M. A. 1994. The skull of *Erlicosaurus andrewsi*, a late Cretaceous ‘Segnosaur’ (Theropoda, Therizinosauridae) from Mongolia. *American Museum Novitates* 3115: 1–39.

Colbert, E. H. 1989. The Triassic dinosaur *Coelophysis*. *Museum of Northern Arizona Bulletin* 57: 1–174.

Colbert, E. H. and Russell, D. A. 1969. The small Cretaceous dinosaur *Dromaeosaurus*. *American Museum Novitates* 2380: 1–49.

Coria, R. A. and Salgado, L. 1998. A basal Abelisauria Novas, 1992 (Theropoda-Ceratosauria) from the Cretaceous of Patagonia, Argentina. *Gaia* 15: 89–102.

Coria, R. A. and Currie, P. J. 2006. A new carcharodontosaurid (Dinosauria, Theropoda) from the Upper Cretaceous of Argentina. *Geodiversitas* 28 (1): 71–118.

Currie, P. J. 1995. New information on the anatomy and relationships of *Dromaeosaurus albertensis* (Dinosauria: Theropoda). *Journal of Vertebrate Paleontology* 15 (3): 576–591.

Currie, P. J. 2003. Cranial anatomy of tyrannosaurid dinosaurs from the Late Cretaceous of Alberta, Canada. *Acta Palaeontologica Polonica* 48 (2): 191–226.

Currie, P. J. 2006. On the quadrate of *Sinraptor dongi* (Theropoda: Allosauroidea) from the Late Jurassic of China. *Mesozoic and Cenozoic Vertebrates and Paleoenvironments. Tributes to the career of Prof. Dan Grigorescu*: 111–115.

Currie, P. J. and Carpenter, K. 2000. A new specimen of *Acrocanthosaurus atokensis* (Theropoda, Dinosauria) from the Lower Cretaceous Antlers Formation (Lower Cretaceous, Aptian) of Oklahoma, USA. *Geodiversitas* 22 (2): 207–246.

Dal Sasso, C. and Maganuco, S. 2011. *Scipionyx samniticus* (Theropoda: Compsognathidae) from the Lower Cretaceous of Italy: osteology, ontogenetic assessment, phylogeny, soft tissue anatomy, taphonomy and palaeobiology. *Memorie della Società Italiana di Scienze Naturali e del Museo Civico di Storia Naturale di Milano* 37 (1): 1–281.

Eddy, D. R. and Clarke, J. A. 2011. New information on the cranial anatomy of *Acrocanthosaurus atokensis* and its implications for the phylogeny of Allosauroidea (Dinosauria: Theropoda). *PLoS ONE* 6 (3): e17932.

Gilmore, C. W. 1920. Osteology of the carnivorous Dinosauria in the United State National museum: with special reference to the genera *Antrodemus* (*Allosaurus*) and *Ceratosaurus*. *Bulletin of the United States National Museum* 110: 1–159.

Hendrickx, C. and Buffetaut, E. 2008. Functional interpretation of spinosaurid quadrates (Dinosauria: Theropoda) from the Mid-Cretaceous of Morocco. *56th Annual Symposium of Vertebrate Palaeontology and Comparative Anatomy. Dublin (September 2nd-6th 2008)*: 25–26.

Holliday, C. M. 2009. New insights into dinosaur jaw muscle anatomy. *The Anatomical Record: Advances in Integrative Anatomy and Evolutionary Biology* 292 (9): 1246–1265.

Holliday, C. M. and Witmer, L. M. 2008. Cranial kinesis in dinosaurs: intracranial joints, protractor muscles, and their significance for cranial evolution and function in diapsids. *Journal of Vertebrate Paleontology* 28 (4): 1073–1088.

Kobayashi, Y. and Lü, J.-C. 2003. A new ornithomimid dinosaur with gregarious habits from the Late Cretaceous of China. *Acta Palaeontologica Polonica* 48 (2): 235–259.

Kobayashi, Y. and Barsbold, R. 2005. Reexamination of a primitive ornithomimosaur, *Garudimimus brevipes* Barsbold, 1981 (Dinosauria: Theropoda), from the Late Cretaceous of Mongolia. *Canadian Journal of Earth Sciences* 42 (9): 1501–1521.

Kundrát, M. and Janáček, J. 2007. Cranial pneumatization and auditory perceptions of the oviraptorid dinosaur *Conchoraptor gracilis* (Theropoda, Maniraptora) from the Late Cretaceous of Mongolia. *Naturwissenschaften* 94 (9): 769–778.

Lautenschlager, S., Witmer, L. M., Altangerel, P., Zanno, L. E. and Rayfield, E. J. 2014. Cranial anatomy of *Erlikosaurus andrewsi* (Dinosauria, Therizinosauria): new insights based on digital reconstruction. *Journal of Vertebrate Paleontology* 34 (6): 1263–1291.

Li, D., Norell, M. A., Gao, K. Q., Smith, N. D. and Makovicky, P. J. 2010. A longirostrine tyrannosauroid from the Early Cretaceous of China. *Proceedings of the Royal Society B: Biological Sciences* 277 (1679): 183–190.

Madsen, J. H. 1976. *Allosaurus fragilis*: A revised osteology. *Utah Geological Survey Bulletin* 109: 1–177.

Madsen, J. H. and Welles, S. P. 2000. *Ceratosaurus* (Dinosauria, Theropoda): a revised osteology. *Utah Geological Survey, Miscellaneous Publication* 00-2: 1–89.

Maryańska, T. and Osmólska, H. 1997. The quadrate of oviraptorid dinosaurs. *Acta Palaeontologica Polonica* 42 (3): 361–371.

Molnar, R. E. 1991. The cranial morphology of *Tyrannosaurus rex*. *Palaeontographica Abteilung A* 217 (4-6): 137–176.

Molnar, R. E. 1998. Mechanical factors in the design of the skull of *Tyrannosaurus rex* (Osborn, 1905). *Gaia* 15: 193–218.

Norell, M. A. and Hwang, S. H. 2004. A troodontid dinosaur from Ukhaa Tolgod (Late Cretaceous Mongolia). *American Museum Novitates* 3446: 1–9.

Norell, M. A., Clark, J. M., Turner, A. H., Makovicky, P. J., Barsbold, R. and Rowe, T. 2006. A new dromaeosaurid theropod from Ukhaa Tolgod (Ömnögov, Mongolia). *American Museum Novitates* 3545: 1–51.

Norell, M. A., Makovicky, P. J., Bever, G. S., Balanoff, A. M., Clark, J. M., Barsbold, R. and Rowe, T. 2009. A review of the Mongolian Cretaceous dinosaur *Saurornithoides* (Troodontidae: Theropoda). *American Museum Novitates* 3654: 1–63.

Rauhut, O. W. M., Milner, A. C. and Moore-Fay, S. 2010. Cranial osteology and phylogenetic position of the theropod dinosaur *Proceratosaurus bradleyi* (Woodward, 1910) from the Middle Jurassic of England. *Zoological Journal of the Linnean Society* 158 (1): 155–195.

Sadleir, R., Barrett, P. M. and Powell, H. P. 2008. The anatomy and systematics of *Eustreptospondylus oxoniensis*, a theropod dinosaur from the Middle Jurassic of Oxfordshire, England. *Monograph of the Palaeontographical Society, London* 160: 1–82.

Sakamoto, M. 2008. Bite force and the evolution of feeding function in birds, dinosaurs and cats. Ph.D. Dissertation, University of Bristol, Brisol, U.K., 254pp.

Sampson, S. D. and Witmer, L. M. 2007. Craniofacial anatomy of *Majungasaurus crenatissimus* (Theropoda: Abelisauridae) from the Late Cretaceous of Madagascar. *Society of Vertebrate Paleontology Memoir* 8: 32–104.

Sedlmayr, J. C. 2002. Anatomy, evolution, and functional significance of cephalic vasculature in Archosauria. Ph.D. Dissertation, Ohio University, Athens, Ohio, USA, 398pp.

Sereno, P. C. and Novas, F. E. 1994. The skull and neck of the basal theropod *Herrerasaurus ischigualastensis*. *Journal of Vertebrate Paleontology* 13 (4): 451–476.

Sereno, P. C., Martinez, R. N., Wilson, J. A., Varricchio, D. J., Alcober, O. A. and Larsson, H. C. E. 2008. Evidence for avian intrathoracic air sacs in a new predatory dinosaur from Argentina. *PLoS ONE* 3 (9): e3303.

Smith, N. D., Makovicky, P. J., Hammer, W. R. and Currie, P. J. 2007. Osteology of *Cryolophosaurus ellioti* (Dinosauria: Theropoda) from the Early Jurassic of Antarctica and implications for early theropod evolution. *Zoological Journal of the Linnean Society* 151 (2): 377–421.

Tahara, R. and Larsson, H. C. E. 2011. Cranial pneumatic anatomy of *Ornithomimus edmontonicus* (Ornithomimidae: Theropoda). *Journal of Vertebrate Paleontology* 31 (1): 127–143.

Welles, S. P. 1984. *Dilophosaurus wetherilli* (Dinosauria, Theropoda). Osteology and comparisons. *Palaeontographica Abteilung A* 185 (4-6): 85–180.

Witmer, L. M. 1990. The craniofacial air sac system of Mesozoic birds (Aves). *Zoological Journal of the Linnean Society* 100 (4): 327–378.

Zanno, L. E. 2010. Osteology of *Falcarius utahensis* (Dinosauria: Theropoda): characterizing the anatomy of basal therizinosaurs. *Zoological Journal of the Linnean Society* 158 (1): 196–230.
